# Identification of Novel Phage Resistance Mechanisms in *Campylobacter jejuni* by Comparative Genomics

**DOI:** 10.3389/fmicb.2021.780559

**Published:** 2021-12-14

**Authors:** Martine C. H. Sørensen, Yilmaz Emre Gencay, Florian Fanger, Mariana A. T. Chichkova, Mária Mazúrová, Jochen Klumpp, Eva M. Nielsen, Lone Brøndsted

**Affiliations:** ^1^Food Safety and Zoonoses, Department of Veterinary and Animal Sciences, University of Copenhagen, Frederiksberg, Denmark; ^2^Institute for Food, Nutrition and Health, ETH Zürich, Zurich, Switzerland; ^3^Foodborne Infections, Department of Bacteria, Parasites & Fungi, Statens Serum Institut, Copenhagen, Denmark

**Keywords:** bacteriophage, *Campylobacter jejuni*, comparative genomics, phage sensitivity, phage resistance, MLST (multilocus sequence typing), McrBC, restriction modification system

## Abstract

Phages infecting *Campylobacter jejuni* are considered a promising intervention strategy at broiler farms, yet phage sensitivity of naturally occurring poultry isolates is not well studied. Here, we investigated phage sensitivity and identified resistance mechanisms of *C. jejuni* strains originating from Danish broilers belonging to the most prevalent MLST (ST) types. Determining plaque formation of 51 phages belonging to *Fletchervirus* or *Firehammervirus* showed that 21 out of 31 *C. jejuni* strains were susceptible to at least one phage. While *C. jejuni* ST-21 strains encoded the common phase variable *O*-methyl phosphoramidate (MeO*P*N) receptor of the *Fletchervirus* and were only infected by these phages, ST-45 strains did not encode this receptor and were exclusively infected by *Firehammervirus* phages. To identify internal phage resistance mechanism in ST-21 strains, we performed comparative genomics of two strains, CAMSA2002 sensitive to almost all *Fletchervirus* phages and CAMSA2038, resistant to all 51 phages. The strains encoded diverse clustered regularly interspaced short palindromic repeats (CRISPR) spacers but none matched the tested phages. Sequence divergence was also observed in a predicted SspE homolog and putative restriction modification systems including a methyl-specific McrBC endonuclease. Furthermore, when *mcrB* was deleted, CAMSA2038 became sensitive to 17 out of 43 phages, three being *Firehammervirus* phages that otherwise did not infect any ST-21 strains. Yet, 16 phages demonstrated significantly lower efficiencies of plating on the *mcrB* mutant suggesting additional resistance mechanism still restricting phage propagation in CAMSA2038. Thus, our work demonstrates that *C. jejuni* isolates originating from broilers may have acquired several resistance mechanisms to successfully prevent phage infection in their natural habitat.

## Introduction

*Campylobacter jejuni* is a zoonotic pathogen and the major cause of food borne associated gastroenteritis in the European Union (EU) (European Food Safety Authority [EFSA] and European Centre for Disease Prevention and Control [ECDC], 2019). Despite the implementation of numerous intervention strategies over the years, a decline in human cases of campylobacteriosis in Europe has not been achieved (European Food Safety Authority [EFSA] and European Centre for Disease Prevention and Control [ECDC], 2019). In addition, human campylobacteriosis is a rising problem in the developing countries and associated with an increased mortality rate in children (Liu et al., 2012; Platts-Mills and Kosek, 2014). *C. jejuni* resides as a commensal in poultry and the main transmission route leading to human infection is the consumption of improperly handled or undercooked contaminated poultry meat (Young et al., 2007; Cody et al., 2019; European Food Safety Authority [EFSA] and European Centre for Disease Prevention and Control [ECDC], 2019). Thus, novel interventions such as applying phages for biocontrol in poultry farms have been proposed and are currently being explored (Carvalho C. M. et al., 2010; Kittler et al., 2013; Chinivasagam et al., 2020). Also, the effect of adding phages directly on chicken meat to reduce the number of *C. jejuni* has been investigated (Goode et al., 2003; Zampara et al., 2017). Successful implementation of such strategies requires a better understanding of phage sensitivity of the highly diverse *C. jejuni* originating from the poultry habitat.

The genetic relationship of *Campylobacter* isolates is commonly studied using multilocus sequence typing (MLST) that is based on the genetic variation present in the seven housekeeping genes (*glyA, uncA, pgm, gltA, glnA, aspA*, and *tkt)* (Dingle et al., 2001). MLST and population studies have demonstrated that *C. jejuni* is genetically highly diverse, resulting in a large number of sequence types (STs) that can be further organized and grouped into clonal complexes (Dingle et al., 2001). Source attribution analyses have shown that MLST ST-21 and to a lesser extent ST-45 are a particular problem for human campylobacteriosis in Denmark (Boysen et al., 2014).

Also, the surface of *C. jejuni* is highly variable. Not only are membrane proteins N-linked glycosylated, the bacterium is also surrounded by a capsule comprised of capsular polysaccharides (CPS) and beneath this, a layer of lipo-oligosaccharides (LOS) is present (Guerry and Szymanski, 2008). Furthermore, two polar flagella ensuring motility of the bacterium are also covered by glycans as a result of the *O*-linked glycosylation locus (Guerry and Szymanski, 2008). Since both the CPS and *O*-linked glycosylation loci are genetically highly variable among *C. jejuni* strains, the carbohydrates composing CPS and LOS are also different (Poly et al., 2011; Karlyshev et al., 2013; Hameed et al., 2020). In addition, homopolymeric tracts (usually a stretch of G’s) are present in several genes within these loci, promoting phase variable gene expression (Parkhill et al., 2000; Bayliss et al., 2012; Aidley et al., 2018). For example, transferases that modify backbone carbohydrates of the CPS with *O*-methyl phosphoramidate (MeO*P*N) and *O*-methyl groups are commonly found to be phase variable expressed in *C. jejuni* (McNally et al., 2007; Aidley et al., 2017). Since phase variation is a stochastic process arising from slipped strand mispairing during DNA replication (Van der Woude, 2011), some cells express the transferases whereas others do not, despite carrying the same genetic loci. Thus, phase variable expression of genes within these loci further contribute to the surface diversity of *C. jejuni*. While the genetic content of the CPS and LOS loci show some correlation with the MLST type (Dingle et al., 2001; Hameed et al., 2020), this is not fully investigated.

Bacteriophages infecting *C. jejuni* primarily belong to the *Firehammervirus* (former Cp220likevirus) or *Fletchervirus* (former Cp8unalike virus) genera and are commonly isolated from chickens (cecum and fecal samples) and related environments (Atterbury et al., 2003; Hansen et al., 2007; Carvalho C. et al., 2010; Owens et al., 2013; Hammerl et al., 2014; Janež et al., 2014; Javed et al., 2014; Sørensen et al., 2015). The *Campylobacter* phages are highly conserved within each genus, but share very limited inter-genera homology (Javed et al., 2014). Moreover, the genomes of both *Fletchervirus* and *Firehammervirus* phages are refractory to most restriction enzymes and were recently shown to contain unusual modifications such as the replacement of guanosine with deoxyinosine and deazaguanosine (Javed et al., 2014; Crippen et al., 2019). In terms of receptor recognition, it is currently only known that *Firehammervirus* phages are dependent on motile flagella for infection, but an actual receptor has not yet been identified (Sørensen et al., 2015). In contrast, the *Fletchervirus* phages rely on CPS for infection and the phase variable MeO*P*N modification has been identified as a phage receptor recognized by many of these phages (Sørensen et al., 2011; Holst Sørensen et al., 2012; Gencay et al., 2018). We recently demonstrated that *Fletchervirus* phages encode up to four different receptor binding proteins (RBP1 to RBP4) (Sørensen et al., 2021). While RBP1 is responsible for binding to the common phase variable MeO*P*N receptor, phase variable expression of RBP2 ensures binding to another currently unknown receptor when MeO*P*N is not expressed (Sørensen et al., 2021). Thus, as a counter-resistance mechanism to phase variable expression of the common MeO*P*N receptor, *Fletchervirus* phages encode multiple receptor binding proteins including two that are phase variably expressed creating phenotypically diverse phage populations (Sørensen et al., 2021).

While receptor mutants and altered surface structures are the first line of bacterial defense, other phage resistance mechanisms target the incoming phage DNA such as restriction and modification (R/M) systems (Labrie et al., 2010). Usually nucleobase methylation provides sequence specific modification of the bacterial genome, thus allowing the cognate restriction endonuclease to discriminate and destroy unmodified invading phage DNA (Labrie et al., 2010). Only one such mechanism has been identified in *C. jejuni*, where a phase variable type IIG restriction-modification system provided partial resistance to phages belonging to both *Fletchervirus* and *Firehammervirus* phages (Anjum et al., 2016). Still, *C. jejuni* phage resistance mechanisms have only been investigated in a limited number of studies, mostly demonstrating phase variable expression of surface structures and loss of motility as the responsible factors (Coward et al., 2006; Sørensen et al., 2011; Holst Sørensen et al., 2012; Aidley et al., 2017; Gencay et al., 2018). Thus, mainly phage resistance mechanisms associated with the first step in phage infection, i.e., binding to the *C. jejuni* surface have been identified so far.

The study of phage sensitivity and development of phage resistance in laboratory *C. jejuni* strains is not sufficient to provide a complete picture of phage sensitivity and resistance mechanisms of more diverse poultry isolates of *C. jejuni*. Here, we aim to investigate phage susceptibility and elucidate underlying phage resistance mechanisms in *C. jejuni* strains isolated from Danish poultry belonging to the most prevalent MLST types observed in Denmark by performing comparative genomics.

## Materials and Methods

### Bacterial Strains, Phages and Growth Conditions

All bacterial strains and *C. jejuni* phages are listed in the Supplementary Tables 1, 2, respectively. Thirty-one *C. jejuni* strains were isolated from Danish broiler chickens in 2007–9 and were selected to represent the most frequent sequence types (ST) in this reservoir (Boysen et al., 2014). *C. jejuni* strains were standardly grown on base II agar plates containing 5% calf blood at 41.6°C under microaerobic conditions (6% O_2_, 6% CO_2_, 88% H_2_N_2_). When appropriate chloramphenicol was added to plates at a final concentration of 20 μg/ml. For phage-related work *C. jejuni* strains were routinely cultivated in brain heart infusion (BHI) broth (Oxoid) supplemented with 10 mM MgSO_4_ and 1 mM CaCl_2_ (CBHI).

### Phage Propagation, Titration and Host Range Determination by Spot Assays

Phages were propagated and titrated as previously described (Gencay et al., 2017; Sørensen et al., 2017). Briefly, for the phage propagation, the plate lysis method was performed using corresponding *C. jejuni* propagation strains and original phage stocks. Propagated phage stocks were stored at 4°C. Phage titration (enumeration) and host range analyses were performed using spot assays (plaque assays) on bacterial lawns. Briefly, *C. jejuni* strains were standardly grown overnight, harvested into CBHI and adjusted to an optical density at 600 nm (OD_600_) of 0.35. Bacterial cultures were then incubated for 4 h at 41.6°C under microaerobic conditions before 500 μl was mixed with 5 ml NZCYM overlay agar (NZCYM broth [Sigma], 0.6% agar) tempered to 45°C and subsequently poured onto premade NZCYM basal agar plates (1.2% agar, 10 μg/ml vancomycin). Plates were then dried for 45 min in a flow hood. Phage stocks were tenfold serial diluted in SM buffer (100 mM NaCl, 8 mM MgSO_4_, 50 mM Tris-HCl, pH 7.5) up to 10^–7^, and three aliquots of 10 μl of the undiluted stock (10^0^) and each serial dilution were spotted on the bacterial lawns made with relevant *C. jejuni* strains. Plates were incubated for 18–24 h at 41.6°C under microaerobic conditions. Following incubation plaques were counted and the mean plaque forming units per ml (pfu/ml) was calculated. For the host range analyses, all experiments were performed in duplicate and the data represent the mean of two or three independent experiments.

### Genome Sequencing, Assembly and Comparative Genomics

Selected *C. jejuni* strains isolated from broiler chickens were genome sequenced using the Illumina platform (Illumina HiSeq) and assembled using CLC genomics workbench version 9.0.1. Average coverage of the genomes ranged from 80-fold to 269-fold. The genomes were annotated using the NCBI Prokaryotic Genome Annotation Pipeline (PGAP). CPS loci were fully assembled using CLC genomics workbench version 9.0.1 by mapping reads to complete *C. jejuni* CPS reference sequences downloaded from GenBank. A CPS reference sequence was identified by blasting (BLASTN) annotated contigs containing CPS genes against all *C. jejuni* genomes available in the NCBI database and choosing the closest matching complete CPS sequence. Fully assembled CPS loci were subsequently confirmed by mapping sequencing reads and annotated using RASTtk (Brettin et al., 2015). The complete annotated CPS loci can be found as an appendix at Mendeley Data: https://data.mendeley.com/datasets/2n9r622g8d/1. Comparison of complete CPS loci was illustrated using Easyfig (Sullivan et al., 2011).

CAMSA2002 and CAMSA2038 were additionally sequenced on a PacBio RS II device (Pacific Biosciences, Menlo Park, CA, United States) using P6/C4 chemistry. One SMRT cell was used for each *C. jejuni* genome. Average coverage for CAMSA2002 was 535-fold while the average coverage for CAMSA2038 was 820-fold. Complete *de novo* genomes of these two strains were assembled using SMRT Analysis version 2.3 and the HGAP3 algorithm. Annotation was performed using the NCBI Prokaryotic Genome Annotation Pipeline (PGAP).

Comparative genomics and McrBC protein sequence alignment was performed using the CLC genomics main workbench 20 and the Whole Genome Alignment plugin. Conserved domains in selected hypothetical protein sequences were identified using InterPro (Mitchell et al., 2019) and HHpred (Zimmermann et al., 2018). Pairwise comparisons between divergent genes were further conducted using BLASTN available at NCBI.

### PCR and Sequencing of PolyG Regions in *cj1421*, *cj1422*, and *cj1426* Homologs

PolyG regions in genes homologous to *cj1421*, *cj1422*, and *cj1426* from *C. jejuni* NCTC11168 were amplified and sequenced (Sanger sequencing) using the primers cj1421F and cj1421R (*cj1421*), cj1422F and cj11422R (*cj1422*) and cj1426F and cj1426R (*cj1426*). Primer sequences are listed in Supplementary Table 3.

### Phage Adsorption Assay

Phage adsorption assays were performed as previously described with minor modifications (Baldvinsson et al., 2014). Briefly, *C. jejuni* were grown overnight on BA plates and harvested in CBHI. Cells were pelleted by centrifugation at 6,000 × *g* for 5 min and re-suspended in CBHI. This washing procedure was repeated twice (total number of washes, three), and the final cell suspension was adjusted to an OD_600_ of 0.4. Bacteriophages were added to the bacterial suspension at a final concentration of 10^6^ PFU/ml (multiplicity of infection [MOI] of approximately 0.0025) and incubated at 37°C with gentle shaking (50–80 rpm) for a total of 90 min. Samples containing free-phages were collected at 0, 15, 30, 60, and 90 min, filtered through an 0.22-μm sterile syringe filter (Millipore), and stored at 4°C until enumeration (plaque assay). Experiments were repeated twice and for each experiment, the calculated phage pfu/ml at time zero was designated as 100% free phages. The percentage of free phages was calculated for the remaining time points according to time zero. All experiments were performed in duplicate and the data represent the mean percentages of free phages and standard deviations thereof from the two independent experiments.

### Motility Assay

Motility assays were performed as previously described (Sørensen et al., 2011). Briefly, *C. jejuni* strains were grown under standard conditions for 18–24 h, harvested into BHI and adjusted to an OD_600_ of 0.1. One microliter of the bacterial suspension was then placed in the center of five to six Heart infusion broth (HIB) (Difco) 0.25% agar plates that had been pre-dried for 45 min in a flow hood. Plates were then incubated under standard growth conditions and growth zones demonstrating movement in the soft agar were measured after 24 h. Measurements and standard deviations represent the mean counts from two independent experiments.

### Construction of *mcrB* Deletion Mutant in *C. jejuni* CAMSA2038

A deletion mutant of *mcrB* in CAMSA2038 was constructed by replacing most of *mcrB* (203 bp to 2009 bp) with a cat cassette by homologous recombination. The vector construct used for homologous recombination was created by fusing three PCR amplicons as described below using In-fusion cloning (Takara). A PCR fragment (778 bp) of the flanking region upstream the *mcrB* deletion was amplified from *C. jejuni* CAMSA2038 using the primers FLOR06_OH2_F2 and FLOR06_OH2_R2. A PCR fragment (878 bp) of the flanking region downstream the *mcrB* deletion was amplified using the primers FLOR06_OH1_F4 and FLOR06_OH1_R3. The *cat* gene (723 bp) was amplified from pRY109 (Yao et al., 1993) using the primers FLOR06_Cat1_fwd and FLOR06_Cat2_rev. The *cat* gene was then inserted between the upstream and downstream *mcrB* deletion fragments and simultaneously inserted into the pET28a+ vector (Novagen) using the In-Fusion kit according to the manufacturer’s instructions. The resulting plasmid pFLOR06 was transformed into *E. coli* Stellar™ Competent Cells (Takara) according to manufacturer’s instructions and verified by sequencing. Purified pFLOR06 plasmid was transformed into *C. jejuni* CAMSA2038 by electroporation to allow homologous recombination of the flanking *mcrB* regions to create the CAMSA2038Δ*mcrB*. The mutant was verified by PCR and sequencing using the primers FLOR06_UP1_fwd, FLOR06_UP2_fwd, and FLOR06_DOWN1_rev. Plasmids and primers are listed in Supplementary Table 3.

### Statistical Analyses

Data in the graphs show the mean values of two independent experiments with standard deviations depicted as error bars. When appropriate one-way ANOVA (GraphPad Prism 9.2.0) was used to assess if differences were statistical significant.

## Results

### Multilocus Sequence Typing ST-Type of *Campylobacter jejuni* Correlate With Sensitivity to Specific Phage Genera

**FIGURE 1:**
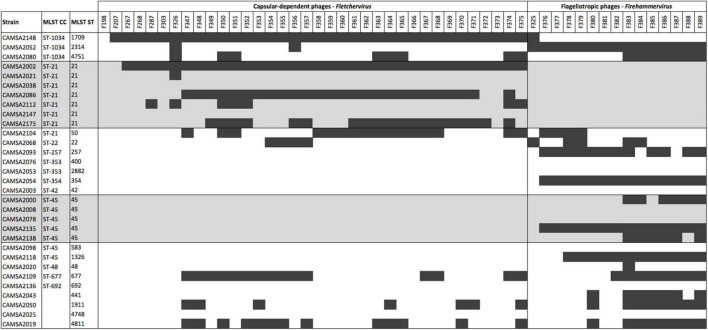
Phage host range analysis of *C. jejuni* broiler isolates characterized by MLST typing. MLST ST-21 and ST-45 isolates are highlighted in gray. Black bars indicate plaque formation detected. CC: clonal complex, ST: sequence type.

To identify putative novel phage resistance mechanisms in *C. jejuni*, we obtained a collection of 31 strains from Statens Serum Institute, which were isolated from broilers in Denmark between 2007 and 2009 and had previously been characterized by MLST typing. These strains represent the ST-types commonly found in broilers in Denmark and included several strains of the two dominant ST-types, ST-21 and ST-45. We then determined phage susceptibility by screening these strains for plaque formation using our collection of 51 *C. jejuni* phages belonging to either *Fletchervirus* or *Firehammervirus*. This host range analysis showed that 21 strains belonging a variety of STs were infected by at least one phage and most of the strains were sensitive to several phages. In contrast, ten strains belonging to different ST-types were completely resistant to all 51 phages (Figure 1). Most ST’s showed a mixed pattern of phage sensitivity, however interestingly, the phage sensitive ST-21 strains were only infected by *Fletchervirus* phages dependent on the CPS for infection. On the contrary, strains belonging to ST-45 were only infected by *Firehammervirus* phages. While our *Fletchervirus* phages are all dependent on the CPS for infection, the *Firehammervirus* are flagellotropic phages dependent on motile flagella (Sørensen et al., 2015). Thus, our results suggest a correlation between sensitivity toward a specific phage genus and the two major *C. jejuni* MLST types, ST-21 and ST-45, often associated with human disease.

### *Campylobacter jejuni* ST-21 Strains Encode the Common *O*-Methyl Phosphoramidate Phage Receptor Recognized by *Fletchervirus* Phages Whereas the ST-45 Strains Do Not

**FIGURE 2:**
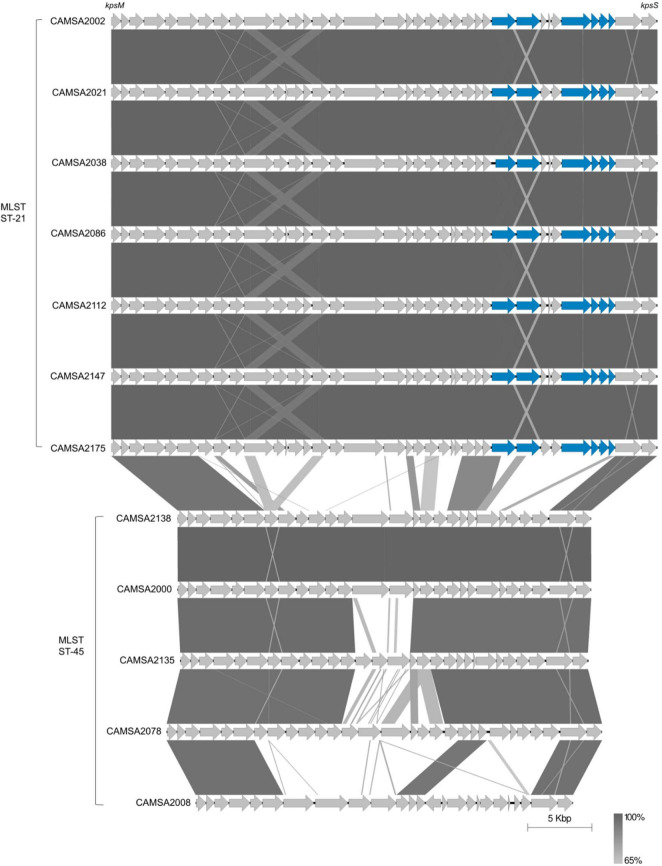
Comparison of CPS loci of ST-21 and ST-45 *C. jejuni* strains. CPS loci are compared using Easyfig (Sullivan et al., 2011). Genes are indicated by gray arrows except for MeO*P*N biosynthesis and transferase genes that are shown as blue arrows. Complete CPS loci sequences can be found at Mendeley Data: https://data.mendeley.com/datasets/2n9r622g8d/1 or are derived from the following: CAMSA2002: GenBank accession no. CP071460.1, CAMSA2038: GenBank accession nos. CP071459.1 and CAMSA2138: GenBank accession no. NZ_QELH01000003.1.

To further investigate the genetic relationship between ST-21 and ST-45, we genome sequenced all strains belonging to these MLST types. As *Fletchervirus* phages are dependent on CPS for infection, we fully assembled and performed comparative genomics of the CPS loci encoded by the ST-21 and ST-45 strains (Figure 2). Our analysis demonstrated that all ST-21 strains encoded identical CPS loci typical of the Penner serotype HS2 group, similar to the CPS locus of the well-characterized *C. jejuni* NCTC11168 strain. The HS2 CPS locus encodes all biosynthesis genes needed to produce the MeO*P*N modification and two MeOPN transferases *cj1421* and *cj1422* responsible for attaching MeO*P*N to Gal*f*NAc and heptose residues in the CPS, respectively. We previously demonstrated that MeO*P*N forms a receptor recognized by several *Fletchervirus* phages both in *C. jejuni* strains NCTC11168 and NCTC12662 (Sørensen et al., 2011; Holst Sørensen et al., 2012; Gencay et al., 2018). Thus, all ST-21 strains encode the common MeO*P*N phage receptor of the *Fletchervirus* phages. On the contrary, the ST-45 strains encode diverse CPS loci, but interestingly no MeO*P*N biosynthesis or transferase genes were found. These results show that the common MeO*P*N phage receptor is not present in any of the ST-45 strains, explaining their resistance toward the CPS-dependent *Fletchervirus* phages.

### Phase Variable Expression of Capsular Polysaccharide Modifications Is Not Responsible for Phage Resistance in ST-21 Strains CAMSA2038 and CAMSA2147 Toward *Fletchervirus* Phages

**FIGURE 3:**
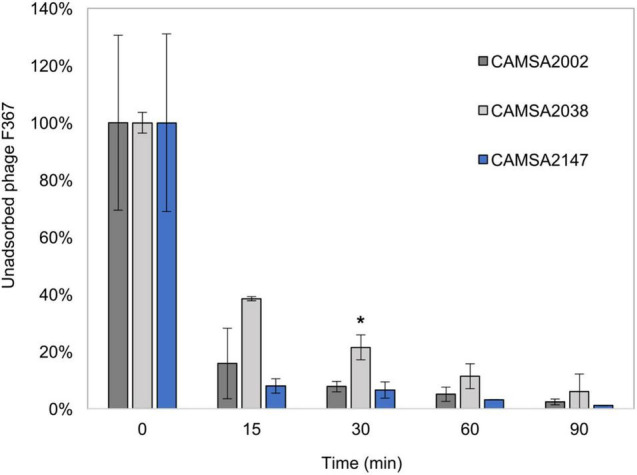
Phage F367 adsorption assay with CAMSA2002, CAMSA2038, and CAMSA2147. Unabsorbed phage F367 is illustrated in percentage over time when incubated with *C. jejuni* strains CAMSA2002 (phage sensitive), CAMSA2038 (phage resistant), and CAMSA2147 (phage resistant). The data represent the mean values and standard deviations from two independent experiments. *Significant different F367 absorption levels (one-way ANOVA, *P*-value 0.0274) was observed only after 30 min when comparing CAMSA2038 (mean: 21%) to CAMSA2002 (mean: 8%).

Although all ST-21 strains encode the common MeO*P*N phage receptor, three of these strains (CAMSA2021, CAMSA2038, and CAMSA2147) are resistant to phage infection (Figure 1). We previously showed that phase variable expression of the MeO*P*N transferases *cj1421* and *cj1422* and the 6-*O*-Me transferase *cj1426* lead to rapid phage resistance development in strain NCTC11168 (Sørensen et al., 2011; Holst Sørensen et al., 2012; Aidley et al., 2017). This was caused by variations in the N-terminal polyG tracts turning the expression of these genes either off (*cj1421*) or on (*cj1422* and *cj1426*) promoting the loss or masking of the MeO*P*N receptor, respectively. To investigate if phase variation could be involved in generating phage resistance in these three ST-21 strains, we compared the polyG tract length in the c*j1421, cj1422*, and *cj1426* homologs with sequencing data obtained following PCR amplification of polyG tract regions in these genes (Table 1). As a control, we also included the highly phage sensitive CAMSA2002 strain in our analysis. For all strains, varying polyG tracts length were detected, suggesting mixed populations with some cells expressing the transferases while others did not. Overall, expression of *cj1421* was mostly turned on in all four strains leading to expression of the MeO*P*N receptor attached to GalfNAc. In contrast, variable tract lengths were detected in *cj1422* and *cj1426* turning expression of the genes both on and off, hence the presence of MeO*P*N attached to Hep and the 6-*O*-Me group varied. Thus, these results indicate that phase variable expression of surface structures leading to loss or masking of the MeO*P*N receptor could not be solely responsible for the observed phage resistant phenotypes. To further investigate if phage resistance was associated with lack of phage binding to the surface of the phage resistant ST-21 strains, we used phage F367 as a representative *Fletchervirus* and determined adsorption to CAMSA2002 (phage sensitive), CAMSA2038 (phage resistant) and CAMSA2147 (phage resistant). Our data showed that phage F367 indeed bind to both resistant strains (CAMSA2038 and CAMSA2147) at levels comparable to the phage sensitive CAMSA2002 (Figure 3). Thus, our data demonstrate that phage resistance toward *Fletchervirus* in CAMSA2038 and CAMSA2147 is not associated with lack of phage binding due to phase variable expression of surface structures and involves potentially novel internal phage resistance mechanisms.

**TABLE 1:** PolyG tract lengths in phase variable CPS genes previously associated with phage resistance in *C. jejuni* as detected during genome sequencing or after complete CPS locus assembly and following PCR amplification.

*C. jejuni* strain	Phage sensitivity	Origin of polyG tract	PolyG tract length and gene expression state (on/off)		
			MeO*P*N-GalfNAc transferase *cj1421* homolog (gene)	MeO*P*N-Hep transferase *cj1422* homolog (gene)	6-*O*-Me transferase *cj1426* homolog (gene)
CAMSA2002	Sensitive	Genome sequence	*(DDR89_07185)* 9 G’s - on	*(DDR89_07190)* 9 G’s - on	*(DDR89_07210)* 9 G’s – on
		PCR	9 G’s - on	10 G’s - off	*^a^*ND
CAMSA2021	Resistant	Complete CPS locus assembly	(*CAMSA2021_28)* 9 G’s - on	(*CAMSA2021_27)* 9 G’s - on	(*CAMSA2021_23)* 10 G’s - on
		PCR	9 G’s - on	10 G’s - off	10 G’s - on
CAMSA2038	Resistant	Genome sequence	(*DDV75_07200)* 9 G’s - on	*(DDV75_07205)* 8 G’s - off	(*DDV75_07225)* 10 G’s - on
		PCR	10 G’s - off	9 G’s - on	10 G’s - on
CAMSA2147	Resistant	Complete CPS locus assembly	(*CAMSA2147_28)* 9 G’s - on	(*CAMSA2147_27)* 9 G’s - on	(*CAMSA2147_22, CAMSA2147_23)* 8 G’s - off
		PCR	9 G’s - on	9 G’s - on	11 G’s - off

*^a^Could not be determined.*

### Motility of ST-21 and ST-45 Strains Does Not Correlate With Sensitivity Toward *Firehammervirus* Phages

While the receptor recognized by *Firehammervirus* phages has not been identified, it was previously demonstrated that lack or reduced motility can lead to phage resistance in *C. jejuni* toward this group of phages (Coward et al., 2006; Sørensen et al., 2015). Since all ST-21 strains are resistant to all *Firehammervirus* phages, we compared the motility of the ST-21 strains with motility patterns observed for the ST-45 that are only infected by this group of phages. However, even though motility varied between strains, the motility of ST-21 strains was comparable to that observed for strains belonging to ST-45 (Supplementary Figure 1). Thus, absence of or reduced motility is not responsible for *Firehammervirus* phage resistance observed in the ST-21 strains. To further investigate if other differences related to flagella biosynthesis could play a role in *Firehammervirus* phage resistance, we compared the flagellar *O*-linked glycosylation loci of the ST-21 and ST-45 strains. Similar to our results obtained from the comparison of the CPS loci, the flagellar *O*-linked glycosylation loci of all the ST-21 strains was similar to the *O*-linked glycosylation encoded by *C. jejuni* NCTC11168, whereas this locus differs among the ST-45 strains (data not shown). Since not much is known about the receptor of *Firehammervirus*, we could not conclude if specific components related to flagella synthesis and glycosylation was responsible for the differences in *Firehammervirus* sensitivity of the ST-21 and ST-45 strains. However, our results show that motility *per se* was not impaired in any of the strains.

### The Genomes of Phage Sensitive CAMSA2002 and Phage Resistant CAMSA2038 Only Differ in Few Genes

**FIGURE 4:**
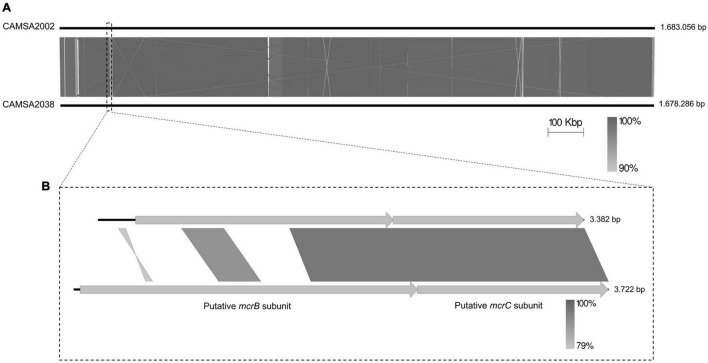
Whole genome comparison of CAMSA2002 and CAMSA2038. **(A)** The genome sequences are depicted as solid lines and the genome sizes are listed in bp. **(B)** Alignment of *mcrBC* genes encoded by CAMSA2002 and CAMSA2038 demonstrating divergence in the N-terminal region responsible for DNA binding. Comparisons are performed using Easyfig (Sullivan et al., 2011).

To identify possible genetic changes responsible for phage resistance in the ST-21 strain CAMSA2038, we performed comparative genomics of CAMSA2038 with the phage sensitive ST-21 CAMSA2002. Overall, we found that the genomes were highly conserved over the entire length on the chromosome including the CPS and *O*-linked glycosylation locus (99.57 average nucleotide identity, 99.11 alignment percentage) (Figure 4A). In addition, the phage sensitive CAMSA2002 contains a plasmid with high sequence similarity to the pTet plasmid of *C. jejuni* strain 81–176 (Supplementary Figure 2), a plasmid encoding tetracycline resistance as it contains a *tetO* gene (Bacon et al., 2000). The pTet plasmid found in CAMSA2002 (38.0 kb) is, however, substantially smaller compared to pTet in 81–176 (45.2 kb) and it does not encode the *tetO* gene. Interestingly, only few genes differed between the CAMSA2002 and CAMSA2038 when not considering SNP mutations (Table 2). Also, sequence divergence was detected in the clustered regularly interspaced short palindromic repeats (CRISPR) region of these two strains. In the following, we will present the major genetic differences detected in the phage sensitive CAMSA2002 and phage resistant CAMSA2038.

**TABLE 2:** Divergent genes found at corresponding genomic positions in CAMSA2002 and CAMSA2038.

Phage sensitive CAMSA2002			Phage resistant CAMSA2038			Similarity		
Gene	Position	Product	Gene	Position	Product	Query cover	Similarity	*E*-value
*DDR89_00040*	12.642–14.393	DUF262 domain-containing protein	*DDV75_00040*	12.653–14.344	DUF262 domain-containing protein	No significant similarity		
*DDR89_00180*	46.404–50.144	*Eco*57I restriction-modification methylase domain-containing protein	*DDV75_00180*	46.521–50.294	*Eco*57I restriction-modification methylase domain-containing protein	88%	94.5%	0.0
*DDR89_00185*	50.147-52.291	AAA family ATPase	*DDV75_00185*	50.693–52.141	Pentapeptide repeat-containing protein	No significant similarity		
*DDR89_00190*	52.276–52.788	Hypothetical protein				Not found in CAMSA2038		
*DDR89_00700*	141.547–143.345	AAA family ATPase	*DDV75_00695*	140.472–142.823	McrB family protein	54%	95.6%	0.0
*DDR89_03100*	588.738–592.166	Hypothetical protein	*DDV75_03095*	587.945–588.988	Type I restriction enzyme HsdR N-terminal domain-containing protein	No significant similarity		
*DDR89_05110*	983.669–987.490	N-6 DNA methylase	*DDV75_05120*	980.543–984.562	N-6 DNA methylase	84%	99.7%	0.0
			*DDV75_06135*	1.187.171–1.187.380	4-oxalocrotonate tautomerase family protein	Not found in CAMSA2002		
*DDR89_06630*	1.275.681–1.276.898	DUF2920 family protein	*DDV75_06645*	1.272.961–1.273.551	DUF2920 family protein	37%	97.0%	0.0
–	1.496.383–1.497.142	CRISPR region	–	1.492.448–1.493.011	CRISPR region	100%	88.1%	3e-143
*DDR89_08675*	1.674.475–1.675.119	Outer membrane beta-barrel protein	*DDV75_08685*	1.670.356–1.671.000	Outer membrane beta-barrel protein	No significant similarity		

### Clustered Regularly Interspaced Short Palindromic Repeats Is Not Associated With Phage Resistance in CAMSA2038

Sequence analysis of the CRISPR region identified more spacers in the phage sensitive CAMSA2002 strain (six spacers) compared to the phage resistant CAMSA2038 (three spacers) (Table 3). Only one spacer sequence was present in both CAMSA2002 and CAMSA2038 whereas the sequences of the remaining spacers differed between the strains (Table 3). The identical spacer shows 100% similarity to phage DA10, an unclassified phage belonging to the *Myoviridae* family unrelated to the *Fletchervirus* and *Firehammervirus* (Hooton et al., 2020). Furthermore, this spacer sequence also showed a match (28 out of 30 bp) to gene *PC5_00034* (hypothetical protein) encoded by *Campylobacter* phage PC5 belonging to the *Fletchervirus* genus (Janež et al., 2014). However, the *PC5_00034* gene is only found in the PC5 and DA10 genomes, and not present in any other currently available *Fletchervirus* phage genomes. Another spacer sequence found in the phage sensitive CAMSA2002 strain showed some sequence similarity to a conserved putative phosphatidyl-serine decarboxylase encoded by all *Fletchervirus* phages (Table 3). But the similarity was rather weak (23–25 bp out of 30 bp) explaining why this spacer does not protect against *Fletchervirus* phage infection, as CAMSA2002 is sensitive to 34 phages belonging to this genus. The remaining spacers found in CAMSA2038 and two spacers in CAMSA2002 show 100% sequence similarity to the genome of phage DA10. These findings show that CAMSA2002 and CAMSA2038 mainly contain sequences originating from phage DA10 in their CRISPR array. In conclusion, *in silico* analysis and host range data support that CRISPR is not responsible for the phage resistant nature of CAMSA2038.

**TABLE 3:** CRISPR region comparison of CAMSA2002 and CAMSA2038 and spacer sequence matches to publicly available *Campylobacter* phage genomes.

*C. jejuni* strain	CRISPR spacer sequence	*Campylobacter* phage genome match (GenBank accession no.)	Position in phage genome	Match identities	*E*-value
CAMSA2002	CTACAAGAATGAGGATGATGATATTTTACA^a)^	DA10 (MN530981.1)	30585-30556	30/30 bp (100%)	3e-6
		PC5 (KX229736.1)	24059-24088	28/30 bp (93%)	0-16
	TCCATTCTCATGAAATATTTAGCCATTATT	PC5 (KX229736.1) PC14 (KX236333) NCTC12673 (FN667788)	69233.69261 112354.112382 77362.77390	25/30 (73%)	2e-06
		vB_CjeM_Los1 (KX879627) F355 (MT863718) F356 (MT863719) F370 (MT863727) F371 (MT863728) F372 (MT863729)	29450.29478 5957.5984 5956.5983 5953.5981 5953.5981 5953.5981	24/30 (70%)	2e-06
		CP30A (NC_018861) CP81 (FR823450) CPX (NC_016562) CP8 (KF148616) F207 (MT863714) F336 (MT863715) F348 (MT863716) F352 (MT863717) F357 (MT863720) F358 (MT863721) F360 (MT863722) F361 (MT863723) F365 (MT863724) F367 (MT863725) F368 (MT863726) F374 (MT863730) F375 (MT863731)	59057.79085 98709.98737 29738.29766 29972.30000 5952.5980 5953.5981 5953.5981 5953.5981 5953.5981 5953.5981 5953.5981 5953.5981 5953.5981 5953.5981 5953.5981 5953.5981 5953.5981	23/30 bp (66%)	8e-06
	TTAAATTTCAAAGATGAGAGTATAGCTAA	DA10 (MN530981.1)	24586-24558	28/29 bp (97%)	0-002
	GTTTCCTTCCTATTTGCTCTATACTCTAAA	None	–		–
	TTAGCAACTTATAATAACTCTAATGTTATT	DA10 (MN530981.1)	3577-3548	30/30 bp (100%)	3e-6
	GCCCCTGCTTTTGATTGAACAAAGCAGCCA	None	–		–
CAMSA2038	TTTCCAAAGTTTCATTAGTTGAATTTAACT	DA10 (MN530981.1)	33283-33312	30/30 bp (100%)	3e-6
	CTACAAGAATGAGGATGATGATATTTTACA^a)^	DA10 (MN530981.1)	30585-30556	30/30 bp (100%)	3e-6
		PC5 (KX229736.1)	24059-24088	28/30 bp (93%)	0-16
	CGCAACTGGTAGCACTTTAACAACTACAGAA	DA10 (MN530981.1)	9061-9031	31/31 bp (100%)	3e-6

*CRISPR repeat sequence: GTTTTAGTCCCTTTTTAAATTTCTTTATGGTAAAAT.*

*^a)^Identical spacer sequence in CAMSA2002 and CAMSA2038.*

### A Putative SspE Homolog Shows Sequence Divergence Between CAMSA2002 and CAMSA2038

Phage sensitive CAMSA2002 and phage resistant CAMSA2038 both encode a DUF262-containing protein (*DDR89_00040* in CAMSA2002, *DDV75_00040* in CAMSA2038) in the same location of the genomes, but the protein sequences show no similarity (Table 2). We therefore conducted a detailed *in silico* analysis to predict putative functions of these two proteins (Supplementary Table 4). In CAMSA2038, the DUF262 is present in the N-terminal of DDV75_00040, but the protein also contains a DUF1524 located in the C-terminal and is predicted by HHpred as a SspE homolog across the entire protein length. In contrast, the corresponding protein DDR89_00040 in CAMSA2002 contains a smaller N-terminal DUF262, but no domains in the C-terminal and thus only the N-terminal region was predicted as a SspE homolog (Supplementary Table 4). Recently, SspE coupled with SspABCD was identified as a phage defense system, where SspE senses and nicks phage DNA not containing phosphorothioate (PT) modifications of the sugar-phosphate DNA backbone produced by SspABCD (Xiong et al., 2020). While the DUF262 encode NTPase and PT sensing activity, the DUF1524 domain is responsible for the nicking endonuclease activity. Thus, both domains are needed to ensure the overall structure, enzymatic and phage resistance activities of SspE (Xiong et al., 2020). It is therefore likely that only CAMSA2038 encodes a true SspE homolog (Supplementary Table 4). Furthermore, SspE only exerts its phage resistance properties when SspABCD is present. It is currently not known if *C. jejuni* DNA contains PT modifications needed for SspE to distinguish and recognize foreign DNA and thus to function. We therefore searched the CAMSA2038 genome for hypothetical proteins containing DUF499 (SspD) and DUF4007 (SspB) domains, however, none such proteins were found. Thus, future experimental verification is needed to show if SspE indeed is associated with phage resistance in CAMSA2038.

### Several Potential Restriction-Modification Systems Differ Between CAMSA2002 and CAMSA2038

Several divergent genes of CAMSA2002 and CAMSA2038 encode putative components of restriction-modification (RM) systems (Table 2). Both CAMSA2002 and CAMSA2038 encode homologs of the RM type IIG system encoded by *cj0031* in *C. jejuni* NCTC11168 (*DDR89_00180* in CAMSA2002, *DDV75_00180* in CAMSA2038). However, significant sequence divergence is observed in the C-terminal region of these genes. *cj0031* encodes both the endonuclease and methylase in a single gene and variations in the C-terminal region has been associated with differences in site-specific methylation (Anjum et al., 2016). Thus, *DDR89_00180* and *DDV75_00180* may methylate different and potential novel sites in CAMSA2002 and CAMSA2038. In addition, the phage resistant CAMSA2038 contains a polyG tract in *DDV75_00180* promoting phase variable expression of the gene.

Other differences include a Type I restriction enzyme HsdR N-terminal domain-containing protein (*DDV75_03095*) encoded by CAMSA2038 that is not found in CAMSA2002 (Table 2). Type I restriction-modification systems are multi-functional complexes encode by three *hsd* genes, *hsdR* (restriction of unmethylated DNA), *hsdM* (modification) and *hsdS* (recognize target sequence) where the gene products form a pentameric R_2_M_2_S complex (Cooper et al., 2017). Both CAMSA2002 and CAMSA2038 encode other *hdsR*, *hsdM* and *hsdS* genes, thus the role of this hypothetical HdsR protein remains unclear.

Finally, CAMSA2038 encodes a putative McrB homolog (*DDV75_00695*) of the McrBC type IV restriction endonuclease system, while CAMSA2002 in the corresponding position on the chromosome encodes a pseudogene (*DDR89_00700*) annotated as a putative AAA family ATPase (Table 2). Comparison of the two genes demonstrated a query coverage of 54% with 96% similarity, suggesting that CAMSA2002 also encodes a putative McrB homolog, yet with a significant different sequence (Figure 4B). Further *In silico* analysis demonstrated that the putative *mcrB* gene in CAMSA2002 contains frameshift mutations thereby resulting in the annotation as a pseudogene. Thus, while both CAMSA2002 and CAMSA2038 encode *mcrC* homologs forming the restriction endonuclease, only CAMSA2038 seems to encode a complete *mcrB* homolog responsible for the DNA binding (Figure 4B). Aligning the *mcrB* genes from CAMSA2002 and CAMSA2038 showed that the far C-terminals were highly conserved, but the N-terminals showed significant sequence divergence (Figure 4B). The N-terminal of the McrB subunit comprises the actual DNA binding region while the C-terminal region contains an AAA + GTPase domain responsible for GTP hydrolysis (Gast et al., 1997; Pieper and Pingoud, 2002; Loenen et al., 2014). Thus, the McrB encoded by CAMSA2038 may recognize a different methylation site or different type of DNA modification as compared to McrB in CAMSA2002. An additional small conserved region was observed near the middle of the two *mcrB* genes (position 965–1235 bp in *DDV75_00695*), suggesting that conservation of this region could be necessary for McrB to establish its function. Further analysis demonstrated that this region covers the beginning of the GTPase domain (data not shown).

In summary, *in silico* analysis indicate that different types of RM systems could be associated with phage resistance in CAMSA2038.

### The McrBC Type IV Restriction Endonuclease Promotes Phage Resistance in *Campylobacter jejuni* Strain CAMSA2038

In *E. coli*, McrBC forms a methyl-specific endonuclease specifically cleaving modified DNA (Zagorskaitė et al., 2018). While McrB homologous are found in several *C. jejuni* strains, not much is known about their function and role in this species (Gardner and Olson, 2012). To investigate the role of McrBC in relation to phage resistance in *C. jejuni* we created a *mcrB* (*DDV75_00695*) deletion mutant in CAMSA2038 thereby rendering the McrBC complex non-functional. We then tested the mutant for phage sensitivity by determining plaque formation using 43 *Fletchervirus* and *Firehammervirus* phages from our collection. Our data showed that CAMSA2038Δ*mcrB* was sensitive to 17 out of the 43 phages tested (Table 4). Fourteen *Fletchervirus* phages were able to infect CAMSA2038 in the absence of *mcrB*. However, in all cases we observed reduced efficiencies of plating as compared to the number of plaques formed on the phage propagation hosts. In addition, plaque formation varied between experiments. Also, three *Firehammervirus* phages (F377, F378, and F379) were able to infect and produce plaques on the *mcrB* mutant. None of the ST-21 strains including CAMSA2038 were otherwise infected by *Firehammervirus* phages. While plaque formation of phage F377 and F379 also demonstrated reduced and variable efficiencies, F378 was able to consistently form plaques at level almost equal to what is observed on its propagation host. Thus, our data demonstrate that McrBC cause phage resistance towards both *Fletchervirus* and *Firehammervirus* phages in *C. jejuni* strain CAMSA2038.

**TABLE 4:** Phage sensitivity of CAMSA2038Δ*mcrB*.

Genus and receptor type	Phage	Propagation host	CAMSA2038	CAMSA2038 Δ *mcrB*
*Fletchervirus*	F347	7,0	–	–
CPS-dependent	F348	8,0	–	–
phages	F349	5,8	–	4,3*
	F350	6,1	–	4,8*
	F351	6,4	–	4,9*
	F352	8,5	–	5,5*
	F353	9,0	–	NC plaques
	F354	8,8	–	NC plaques
	F355	9,0	–	–
	F356	6,9	–	–
	F357	9,7	–	8,2*
	F358	9,5	–	–
	F359	8,1	–	6,2*
	F360	9,2	–	–
	F361	9,4	–	–
	F362	8,4	–	6,5*
	F363	6,9	–	–
	F364	6,9	–	4,7*
	F365	9,0	–	–
	F366	9,1	–	6,8*
	F367	9,4	–	6,7*
	F368	9,2	–	7,5*
	F369	7,8	–	5,3
	F370	9,0	–	–
	F371	9,1	–	–
	F372	9,4	–	–
	F373	8,2	–	–
	F374	6,6	–	–
	F375	9,3	–	–
*Firehammervirus*	F376	9,4	–	–
Flagellotropic	F377	9,3	–	4,8*
phages	F378	9,5	–	8,8
	F379	9,8	–	4,2*
	F380	9,4	–	–
	F381	8,8	–	–
	F382	8,7	–	–
	F383	9,1	–	–
	F384	9,6	–	–
	F385	9,6	–	–
	F386	9,4	–	–
	F387	9,2	–	–
	F388	9,8	–	–
	F389	9,5	–	–

Values represent log10 (pfu/ml). NC, non-countable plaques. –, no lysis or plaques detected. *Plaques detected in 1 or 2 out of three experiments.

## Discussion

Despite implementation of numerous intervention strategies during the last decades, *C. jejuni* remains the major cause of foodborne gastroenteritis in EU as well as in developing countries (Platts-Mills and Kosek, 2014; European Food Safety Authority [EFSA] and European Centre for Disease Prevention and Control [ECDC], 2019). While phage therapy is a promising strategy to reduce *C. jejuni* in primary production and on food, not much is known about phage sensitivity and phage resistance mechanisms in naturally occurring isolates originating from poultry and broilers. Previous studies using laboratory *C. jejuni* strains have shown that phase variable expression of surface structures such as a common phage receptor, and loss of motility cause resistance toward *Fletchervirus* and *Firehammervirus* phages infecting *C. jejuni* (Coward et al., 2006; Sørensen et al., 2011; Holst Sørensen et al., 2012; Aidley et al., 2017; Gencay et al., 2018). In contrast, only a few internal phage resistance mechanisms have been described in *C. jejuni* involving induction of a Mu-like prophage and phase variable expression of a type IIG RM system able to partially restrict invading phage DNA (Scott et al., 2007; Anjum et al., 2016). Here, we determine the phage susceptibility of 31 *C. jejuni* isolates originating from Danish broilers and use comparative genomics to identify several putative phage resistance mechanisms. Furthermore, we provide experimental evidence that the type IV McrBC restriction endonuclease acts as a phage resistance mechanism in *C. jejuni*.

Population studies of *C. jejuni* strains using MLST have demonstrated a large genetic diversity within this species (Dingle et al., 2002). However, the MLST types ST-21 and ST-45 are the two most frequent STs recorded in the pubMLST database (4% each; February 6, 2021)^1^. Despite a large geographical variation in MLST distributions, most studies find that ST-21 and ST-45 are often associated with foodborne gastroenteritis and represent the most prevalent STs both in broilers and human cases of campylobacteriosis (Cody et al., 2012; McCarthy et al., 2012; Griekspoor et al., 2015; Mossong et al., 2016). It is therefore important to investigate the phage susceptibility of particularly *C. jejuni* ST-21 and ST-45 strains originating from the broiler habitat to support successful implementation of phage therapy against *C. jejuni* at farm level. Using 51 phages we found a correlation between sensitivity to a phage genus and the ST-type as our ST-21 strains were only infected by *Fletchervirus* phages, whereas ST-45 strains were exclusively infected by phages belonging to the *Firehammervirus* genus. ST-21 and ST-45 represent two genetically distinct generalist linages that have emerged independently, but both having a wide range of host animals (Sheppard et al., 2014; Yahara et al., 2017). It is thus intriguing to speculate if the two distinct *Campylobacter* phage genera have evolved to specifically target one of these major lineages. Yet, the number of ST-21 (seven) and ST-45 (five) strains included in our analysis was rather limited, thus it will be important to test the phage susceptibility of more such isolates to verify if a correlation indeed exist.

A number of other studies have investigated the phage susceptibility of several diverse *C. jejuni* isolates originating from the chicken/broiler habitat (Loc Carrillo et al., 2005; Hansen et al., 2007; Hammerl et al., 2014; Janež et al., 2014; Nowaczek et al., 2019). In all these studies, several isolates demonstrated complete resistance toward the phages applied, however, often only a limited number of phages were tested, which could explain the high level of resistance. Interestingly, in one study nine phages were screened against 49 *C. jejuni* strains of both human (24 strains) and broiler (25 strains) origin, and while 80% of the strains of human origin were susceptible to phage infection only 44% of the *C. jejuni* strains of broiler origin could be infected by the phages (Janež et al., 2014). We also observed that 10 out of 31 strains were completely resistant to phage infection despite including 51 phages in our analysis. These phage resistant strains belonged to several diverse ST types and were also found within the ST-21 and ST-45 groups such as CAMSA2038 (ST-21). Such observations suggest that the *C. jejuni* strains present in broilers may have acquired several phage resistance mechanisms in order to survive the phage predation in their natural habitat. Thus, this could represent a challenge for the future phage applications targeting *C. jejuni* in broilers and supports the need for investigating underlying resistance mechanisms in these isolates.

Our comparative genomics identified several RM systems that were divergent between the phage sensitive CAMSA2002 and phage resistant CAMSA2038 strains, including the type IV restriction endonuclease McrBC cleaving only modified DNA. While McrBC is widespread in *C. jejuni*, little is known about its function, but it was previously noted that expression of *mcrC* was 1.75-fold upregulated in *C. jejuni* NCTC11168 during infection with the *Fletchervirus* phage NCTC12673 (Sacher et al., 2018). We here experimentally confirm that McrBC of CAMSA2038 acts as a novel phage resistance mechanism in *C. jejuni* targeting phages belonging to both *Fletchervirus* and *Firehammervirus*. The genomes of both *Fletchervirus* and *Firehammervirus* phages are highly modified containing unusual bases, but also other types of DNA modifications may exist due to their refractory nature toward several restriction nucleases (Javed et al., 2014; Crippen et al., 2019). In *E. coli*, McrBC was shown to specifically cleave phage genomes containing 5-hydroxymethycytosine, 5-methylcytosine and N4-methylcystosine (Zagorskaitė et al., 2018). McrB recognizes these modified bases by a base flipping mechanism where cytosine is flipped out of the DNA helix and positioned within the binding pocket of McrB for further discrimination (Sukackaite et al., 2012). In *E. coli*, the pocket size and pocket residues distinguish pyrimidines from purines to pre-select cytosine and is large enough to accommodate cytosine, 5mC, 5hmC, or 4mC, but cannot fit glycosylated derivatives (Sukackaite et al., 2012; Zagorskaitė et al., 2018). Interestingly, the McrB subunit in *C. jejuni* CAMSA2038 (784 aa) is almost double the size of the McrB subunit in *E. coli* K12 (459 aa), mainly due to a larger N-terminal region responsible for DNA binding. It is tempting to speculate that the larger N-terminal in CAMSA2038 creates a different pocket size, pocket residues or structural organization, allowing another type of base discrimination, and consequently different or larger modified bases to be recognized (Zagorskaitė et al., 2018) such as modifications encoded by the *Fletchervirus* and *Firehammervirus* phages. Indeed, it is interesting that McrBC is able to target the DNA of both *Fletchervirus* and *Firehammervirus* phages, as these two genera are highly distinct genetically and the base substitutions already identified are diverse, i.e., deoxyinosine versus deazaguanine (Crippen et al., 2019). This supports that McrBC also in *C. jejuni* may recognize a more common DNA modification found in some phages of both genera. Most type IV RM systems described so far indeed recognize and cleave the common m6A, 5mC, or 5hmC modifications despite demonstrating huge diversity (Loenen et al., 2014), and it is currently unknown if our *C. jejuni* phages contain such DNA modifications. However, the discovery of other type IV RM systems such as the PvuRts1 family and GmrSD that recognize and cleave larger glycosylated bases (Janosi et al., 1994; Bair and Black, 2007; Weigele and Raleigh, 2016) demonstrate that other types of modified DNA certainly can be targeted by this group of enzymes. Especially the observation that our *Firehammervirus* phage F378 showed consistent plaque formation on the CAMSA2038Δ*mcrB* mutant at frequencies comparable to its propagation host, clearly demonstrate McrBC acting as the major defense mechanism in CAMSA2038 against this phage. We are currently investigating DNA modifications and modification motifs present in phage F378 to further elucidate the type of DNA modification recognized by McrBC in *C. jejuni*.

Resistance caused by type I-III RM systems against phage infection is rarely as efficient as preventing the phage from binding such as by altering or removing the phage receptor (Labrie et al., 2010). While these RM systems decrease the chance of productive phage infection, they rarely stop infection completely, as the phage DNA can become modified by host methylases, thus rendering host endonucleases ineffective against the phage progeny (Sneppen et al., 2015). This was observed in *C. jejuni* NCTC11168, where the type IIG RM system only lead to partial resistance against *Fletchervirus* and *Firehammervirus* phages (Anjum et al., 2016). Here, we identified putative type I and II RM systems in the phage resistant *C. jejuni* strain (CAMSA2038) that could play a role in generating phage resistance, including a homolog of the type IIG RM system. Thus, the presence of several RM systems targeting invading phage DNA may have complementary effects to consequently prevent phage infection more effectively as observed for CAMSA2038. This is supported by our observation that most *Fletcherviruses* and *Firehammervirus* showed a much lower efficiency of plating on the *mcrB* mutant in CAMSA2038 compared to their propagation hosts, suggesting that additional factors are still restricting efficient phage replication in CAMSA2038. For many of these phages, we also observed variable results, i.e., plaque formation occurring in some experiments while not in others, in addition to smaller sized plaques being formed on CAMSA2038Δ*mcrB* (data not shown). We have previously observed variation in plaque formation of *Fletchervirus* phages due to phase variable expression of CPS surface structure including the common MeO*P*N phage receptor recognized by this group of phages (Sørensen et al., 2011; Holst Sørensen et al., 2012; Gencay et al., 2018). But, also in CAMSA2038 the type IIG RM system is phase variably expressed due to the presence of a polyG tract as observed in *C. jejuni* strain NCTC11168 (Anjum et al., 2016). Indeed, smaller plaque sizes have been associated with low adsorption rates (Gallet et al., 2011), but could also reflect progeny phages circumventing type I and II RM systems due to methylation by the corresponding host methylases. We previously demonstrated that phase variable expression of CPS surface structures acts as a major defense mechanism against the *Fletchervirus* phages also in the chicken gut (Sørensen et al., 2011; Holst Sørensen et al., 2012; Aidley et al., 2017; Gencay et al., 2018). Yet, we recently discovered that phages belonging to this genus can encode up to four different receptor binding protein which allows these phages to infect *C. jejuni* in the absence of the common phase variable MeO*P*N receptor (Sørensen et al., 2021). This supports the need for complementary phage defense mechanisms in *C. jejuni* to efficiently prevent infection by this group of phages. Resistance toward flagellotropic *C. jejuni* phages has mostly been associated with loss of motility in laboratory settings (Coward et al., 2006; Scott et al., 2007; Baldvinsson et al., 2014). Motility is however, a key feature for establishing colonization of *C. jejuni* in the chicken gut (Young et al., 2007; Epps et al., 2013), thus additional defense mechanism such as McrBC targeting such phages, i.e., *Firehammervirus* are expected to be present in poultry isolates.

In conclusion, our work demonstrates that *C. jejuni* isolates originating from broilers may have acquired several resistance mechanisms to efficiently prevent phage infection in their natural habitat and that the study of such isolates may reveal novel phage resistance mechanisms.

## Data Availability Statement

The datasets presented in this study can be found in online repositories. The names of the repository/repositories and accession number(s) can be found in the Supplementary Material and below: https://data.mendeley.com/datasets/2n9r622g8d/1, Mendeley Data.

## Author Contributions

MS, YG, EN, and LB contributed to the conceptualization and funding acquisition. MS, YG, JK, EN, and LB contributed to the methodology and resources. MS, YG, FF, MC, MM, and JK contributed to the investigation. MS and YG contributed to the formal analysis and supervision. MS and LB contributed to the writing – original draft. All authors contributed to the article and approved the submitted version.

## Conflict of Interest

YG is currently employed by SNIPR Biome. The salary of MS was partially funded by Intralytix, Inc., but the company had no influence on the design of the study nor any impact on the conclusions of the presented work. The remaining authors declare that the research was conducted in the absence of any commercial or financial relationships that could be construed as a potential conflict of interest.

## Publisher’s Note

All claims expressed in this article are solely those of the authors and do not necessarily represent those of their affiliated organizations, or those of the publisher, the editors and the reviewers. Any product that may be evaluated in this article, or claim that may be made by its manufacturer, is not guaranteed or endorsed by the publisher.

## Footnotes

1
https://pubmlst.org/campylobacter
