# Supplementary material for: Identification of Novel Phage Resistance Mechanisms in Campylobacter jejuni by Comparative Genomics
Source: Front Microbiol. 2021 Dec 14;12:780559. doi: 10.3389/fmicb.2021.780559 (PMC8713573; doi:10.3389/fmicb.2021.780559)
Supplement: Supplementary file 1 [file Data_Sheet_1.docx]

Supplementary Material

**
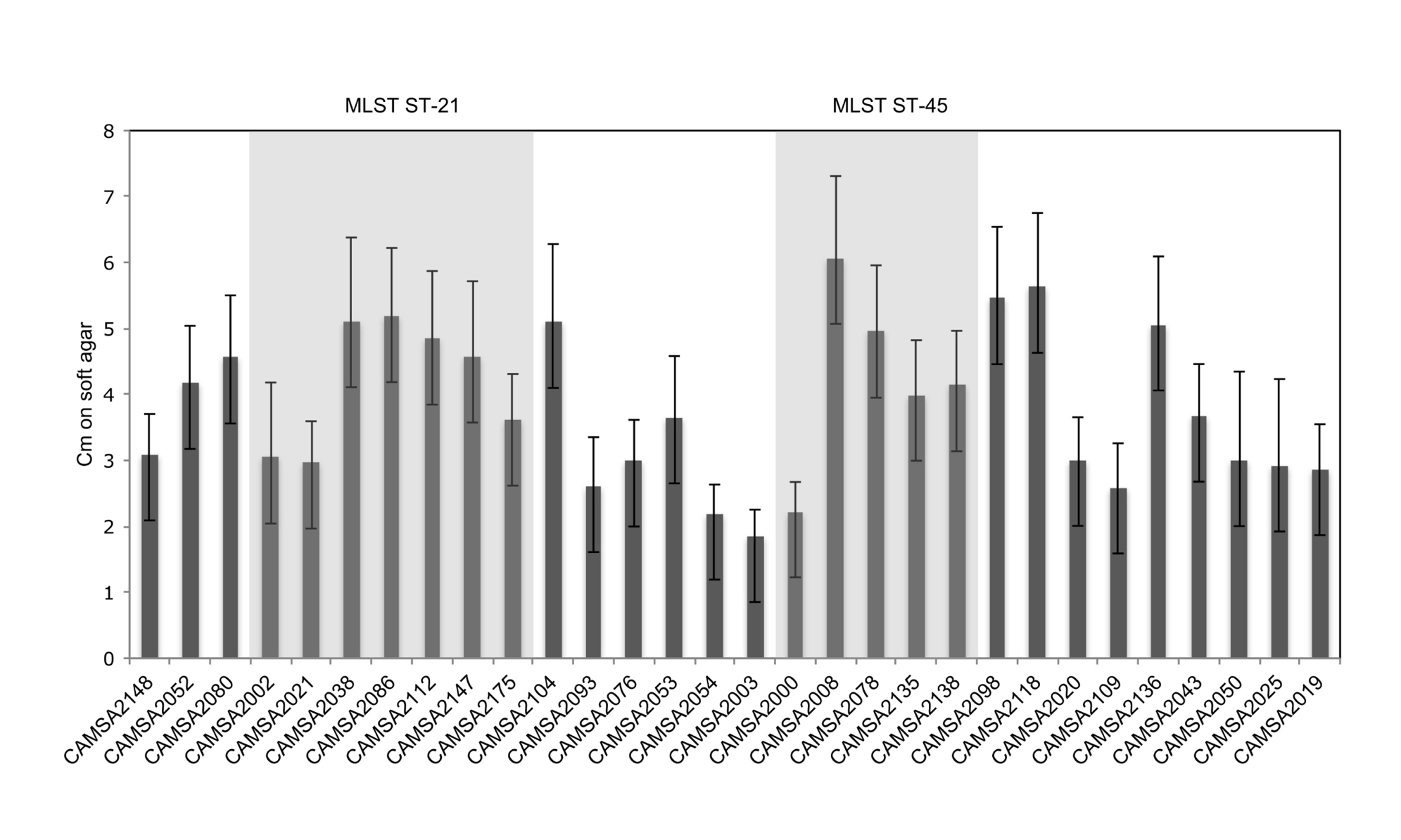
**

**Supplementary Figure 1. Motility profiles of *C. jejuni* CAMSA strains.** Mean values showing diameters of growth zones in cm and standard deviations depicted as error bars are from two independent experiments. MLST ST-21 and ST-45 strains are highlighted in grey.

**Supplementary Figure 2. Comparison of pTet plasmids encoded by *C. jejuni* strain 81-176 and CAMSA2002.** Complete plasmid sequences are compared using Easyfig (Sullivan et al., 2011). Genes are depicted as grey arrows and the *tetO* gene encoding tetracycline resistance is indicated by name.

**Supplementary Table 1.** Bacterial strains.

| ***C. jejuni* strain** | **Isolate no.** | **Source** | **Origin** | **Isolation date** | **MLST**  **CC** | **MLST ST** | **Reference** | **Genbank BioSample no.** |
| --- | --- | --- | --- | --- | --- | --- | --- | --- |
| CAMSA2148 | 2007-55-208 | Broiler,  cloacal swab | DK | 2007-07-19 | ST-1034 complex | 1709 | This study | NA |
| CAMSA2052 | 2008-55-220 | Broiler,  cloacal swab | DK | 2008-07-21 | ST-1034 complex | 2314 | This study | NA |
| CAMSA2080 | 2008-55-439 | Broiler,  cloacal swab | DK | 2008-11-04 | ST-1034 complex | 4751 | This study | NA |
| CAMSA2002 | 2008-55-255 | Broiler,  cloacal swab | DK | 2008-08-18 | ST-21 complex | 21 | This study | SAMN08987254 |
| CAMSA2021 | 2008-55-291 | Broiler,  cloacal swab | DK | 2008-08-06 | ST-21 complex | 21 | This study | SAMN08987255 |
| CAMSA2038 | 2008-55-191 | Broiler,  cloacal swab | DK | 2008-07-14 | ST-21 complex | 21 | This study | SAMN08987256 |
| CAMSA2086 | 2009-70-95-9 | Broiler,  cecum | DK | 2008-05-20 | ST-21 complex | 21 | This study | SAMN08987257 |
| CAMSA2112 | 2007-55-43 | Broiler, cloacal swab | DK | 2007-03-15 | ST-21 complex | 21 | This study | SAMN08987258 |
| CAMSA2147 | 2007-55-205 | Broiler, cloacal swab | DK | 2007-07-17 | ST-21 complex | 21 | This study | SAMN08987259 |
| CAMSA2175 | 2007-55-266 | Broiler, cloacal swab | DK | 2007-08-06 | ST-21 complex | 21 | This study | SAMN08987260 |
| CAMSA2104 | 2009-70-97-5 | Broiler, cecum | DK | 2008-10-01 | ST-21 complex | 50 | This study | SAMN08987261 |
| CAMSA2068 | 2008-55-328 | Broiler, cloacal swab | DK | 2008-09-16 | ST-22 complex | 22 | This study | SAMN08987262 |
| CAMSA2093 | 2009-70-96-7 | Broiler, cecum | DK | 2008-08-04 | ST-257 complex | 257 | This study | NA |
| CAMSA2076 | 2008-55-358 | Broiler, cloacal swab | DK | 2008-10-07 | ST-353 complex | 400 | This study | NA |
| CAMSA2053 | 2008-55-222 | Broiler, cloacal swab | DK | 2008-07-15 | ST-353 complex | 2882 | This study | NA |
| CAMSA2054 | 2008-55-226 | Broiler, cloacal swab | DK | 2008-07-23 | ST-354 complex | 354 | This study | NA |
| CAMSA2003 | 2008-55-256 | Broiler, cloacal swab | DK | 2008-07-30 | ST-42 complex | 42 | This study | NA |
| CAMSA2000 | 2008-55-253 | Broiler, cloacal swab | DK | 2008-07-28 | ST-45 complex | 45 | This study | SAMN08987263 |
| CAMSA2008 | 2008-55-266 | Broiler, cloacal swab | DK | 2008-08-04 | ST-45 complex | 45 | This study | SAMN08987264 |
| CAMSA2078 | 2008-55-379 | Broiler, cloacal swab | DK | 2008-10-10 | ST-45 complex | 45 | This study | SAMN08987265 |
| CAMSA2135 | 2007-55-187 | Broiler, cloacal swab | DK | 2007-07-12 | ST-45 complex | 45 | This study | SAMN08987266 |
| CAMSA2138 | 2007-55-191 | Broiler, cloacal swab | DK | 2007-07-10 | ST-45 complex | 45 | This study | SAMN08987267 |
| CAMSA2098 | 2009-70-96-16 | Broiler, cecum | DK | 2008-08-18 | ST-45 complex | 583 | This study | NA |
| CAMSA2118 | 2007-55-148 | Broiler, cloacal swab | DK | 2007-05-30 | ST-45 complex | 1326 | This study | NA |
| CAMSA2020 | 2008-55-290 | Broiler, cloacal swab | DK | 2008-08-01 | ST-48 complex | 48 | This study | NA |
| CAMSA2109 | 2007-55-23 | Broiler, cloacal swab | DK | 2007-02-16 | ST-677 complex | 677 | This study | SAMN08987268 |
| CAMSA2136 | 2007-55-189 | Broiler, cloacal swab | DK | 2007-07-16 | ST-692 complex | 692 | This study | NA |
| CAMSA2043 | 2008-55-197 | Broiler, cloacal swab | DK | 2008-07-16 | none | 441 | This study | NA |
| CAMSA2050 | 2008-55-217 | Broiler, cloacal swab | DK | 2008-07-21 | none | 1911 | This study | SAMN08987269 |
| CAMSA2025 | 2008-55-301 | Broiler, cloacal swab | DK | 2008-08-19 | none | 4748 | This study | NA |
| CAMSA2019 | 2008-55-289 | Broiler, cloacal swab | DK | 2008-08-07 | none | 4811 | This study | SAMN08987270 |
| **Other *C. jejuni* strains** | **Description** | | | | | | **Reference** | |
| NCTC12658 | Phage propagation host | | | | | | National collection of type cultures | |
| NCTC12662 | Phage propagation host | | | | | | National collection of type cultures | |
| RM1221 | Phage propagation host | | | | | | Fouts et al., 2005 | |
| CAMSA2038*ΔmcrB* | *mcrB* deletion mutant in CAMSA2038, Cam^R^ (20 μg/ml) | | | | | | This study | |

NA: Not applicable, strain not sequenced

**Supplementary Table 2.** *C. jejuni* phages

| **Phage** | ***C. jejuni* host** | **Origin** | **Genus** | **Receptor type** | **Reference** | **GenBank access. no.** |
| --- | --- | --- | --- | --- | --- | --- |
| F198 | NCTC12662 | Broiler intestine | *Fletchervirus* | CPS | Hansen et al., 2007 | NA |
| F207 | NTCT12662 | Duck abattoir | *Fletchervirus* | CPS | Hansen et al., 2007, Sørensen et al., 2021 | MT863714 |
| F267 | NCTC12662 | Broiler abattoir | *Fletchervirus* | CPS | Hansen et al., 2007 | NA |
| F268 | NCTC12662 | Broiler abattoir | *Fletchervirus* | CPS | Hansen et al., 2007 | NA |
| F287 | NCTC12662 | Duck intestine | *Fletchervirus* | CPS | Hansen et al., 2007 | NA |
| F303 | NCTC12662 | Duck abattoir | *Fletchervirus* | CPS | Hansen et al., 2007 | NA |
| F326 | NCTC12662 | Duck intestine | *Fletchervirus* | CPS | Hansen et al., 2007 | NA |
| F347 | NCTC12658 | Free-range chicken fecal sample | *Fletchervirus* | CPS | Sørensen et al., 2015 | NA |
| F348 | NCTC12658 | Free-range chicken fecal sample | *Fletchervirus* | CPS | Sørensen et al., 2015 | MT863716 |
| F349 | NCTC12658 | Free-range chicken fecal sample | *Fletchervirus* | CPS | Sørensen et al., 2015, Sørensen et al., 2021 | NA |
| F350 | NCTC12658 | Free-range chicken fecal sample | *Fletchervirus* | CPS | Sørensen et al., 2015 | NA |
| F351 | NCTC12658 | Free-range chicken fecal sample | *Fletchervirus* | CPS | Sørensen et al., 2015 | NA |
| F352 | NCTC12658 | Free-range chicken fecal sample | *Fletchervirus* | CPS | Sørensen et al., 2015, Sørensen et al., 2021 | MT863717 |
| F353 | NCTC12662 | Free-range chicken fecal sample | *Fletchervirus* | CPS | Sørensen et al., 2015 | NA |
| F354 | NCTC12662 | Free-range chicken fecal sample | *Fletchervirus* | CPS | Sørensen et al., 2015 | NA |
| F355 | NTCT12662 | Free-range chicken fecal sample | *Fletchervirus* | CPS | Sørensen et al., 2015, Sørensen et al., 2021 | MT863718 |
| F356 | NTCT12662 | Free-range chicken fecal sample | *Fletchervirus* | CPS | Sørensen et al., 2015, Sørensen et al., 2021 | MT863719 |
| F357 | NTCT12662 | Free-range chicken fecal sample | *Fletchervirus* | CPS | Sørensen et al., 2015, Sørensen et al., 2021 | MT863720 |
| F358 | NTCT12662 | Free-range chicken fecal sample | *Fletchervirus* | CPS | Sørensen et al., 2015, Sørensen et al., 2021 | MT863721 |
| F359 | NCTC12662 | Free-range chicken fecal sample | *Fletchervirus* | CPS | Sørensen et al., 2015 | NA |
| F360 | NTCT12662 | Free-range chicken fecal sample | *Fletchervirus* | CPS | Sørensen et al., 2015, Sørensen et al., 2021 | MT863722 |
| F361 | NTCT12662 | Free-range chicken fecal sample | *Fletchervirus* | CPS | Sørensen et al., 2015, Sørensen et al., 2021 | MT863723 |
| F362 | NCTC12662 | Free-range chicken fecal sample | *Fletchervirus* | CPS | Sørensen et al., 2015 | NA |
| F363 | NCTC12662 | Free-range chicken fecal sample | *Fletchervirus* | CPS | Sørensen et al., 2015 | NA |
| F364 | NCTC12662 | Free-range chicken fecal sample | *Fletchervirus* | CPS | Sørensen et al., 2015 | NA |
| F365 | NTCT12662 | Free-range chicken fecal sample | *Fletchervirus* | CPS | Sørensen et al., 2015, Sørensen et al., 2021 | MT863724 |
| F366 | NCTC12662 | Free-range chicken fecal sample | *Fletchervirus* | CPS | Sørensen et al., 2015 | NA |
| F367 | NTCT12662 | Free-range chicken fecal sample | *Fletchervirus* | CPS | Sørensen et al., 2015, Sørensen et al., 2021 | MT863725 |
| F368 | NTCT12662 | Free-range chicken fecal sample | *Fletchervirus* | CPS | Sørensen et al., 2015, Sørensen et al., 2021 | MT863726 |
| F369 | NCTC12662 | Free-range chicken fecal sample | *Fletchervirus* | CPS | Sørensen et al., 2015 | NA |
| F370 | NTCT12662 | Free-range chicken fecal sample | *Fletchervirus* | CPS | Sørensen et al., 2015, Sørensen et al., 2021 | MT863727 |
| F371 | NTCT12662 | Free-range chicken fecal sample | *Fletchervirus* | CPS | Sørensen et al., 2015, Sørensen et al., 2021 | MT863728 |
| F372 | NTCT12662 | Free-range chicken fecal sample | *Fletchervirus* | CPS | Sørensen et al., 2015, Sørensen et al., 2021 | MT863729 |
| F373 | NTCT12662 | Free-range chicken fecal sample | *Fletchervirus* | CPS | Sørensen et al., 2015 | NA |
| F374 | NTCT12662 | Free-range chicken fecal sample | *Fletchervirus* | CPS | Sørensen et al., 2015, Sørensen et al., 2021 | MT863730 |
| F375 | NTCT12662 | Free-range chicken fecal sample | *Fletchervirus* | CPS | Sørensen et al., 2015, Sørensen et al., 2021 | MT863731 |
| F325 | NCTC12662 | Duck intestine | *Firehammervirus* | Flagella | Hansen et al., 2007 | NA |
| F376 | RM1221 | Free-range chicken fecal sample | *Firehammervirus* | Flagella | Sørensen et al., 2015 | NA |
| F377 | RM1221 | Free-range chicken fecal sample | *Firehammervirus* | Flagella | Sørensen et al., 2015 | NA |
| F378 | RM1221 | Free-range chicken fecal sample | *Firehammervirus* | Flagella | Sørensen et al., 2015 | NA |
| F379 | RM1221 | Free-range chicken fecal sample | *Firehammervirus* | Flagella | Sørensen et al., 2015 | NA |
| F380 | RM1221 | Free-range chicken fecal sample | *Firehammervirus* | Flagella | Sørensen et al., 2015 | NA |
| F381 | RM1221 | Free-range chicken fecal sample | *Firehammervirus* | Flagella | Sørensen et al., 2015 | NA |
| F382 | RM1221 | Free-range chicken fecal sample | *Firehammervirus* | Flagella | Sørensen et al., 2015 | NA |
| F383 | RM1221 | Free-range chicken fecal sample | *Firehammervirus* | Flagella | Sørensen et al., 2015 | NA |
| F384 | RM1221 | Free-range chicken fecal sample | *Firehammervirus* | Flagella | Sørensen et al., 2015 | NA |
| F385 | RM1221 | Free-range chicken fecal sample | *Firehammervirus* | Flagella | Sørensen et al., 2015 | NA |
| F386 | RM1221 | Free-range chicken fecal sample | *Firehammervirus* | Flagella | Sørensen et al., 2015 | NA |
| F387 | RM1221 | Free-range chicken fecal sample | *Firehammervirus* | Flagella | Sørensen et al., 2015 | NA |
| F388 | RM1221 | Free-range chicken fecal sample | *Firehammervirus* | Flagella | Sørensen et al., 2015 | NA |
| F389 | RM1221 | Free-range chicken fecal sample | *Firehammervirus* | Flagella | Sørensen et al., 2015 | NA |

NA: Not applicable, phage not sequenced.

**Supplementary Table 3: Plasmids and primers.** Overhangs added to primers used for In-Fusion cloning are marked in bold.

| **Plasmid** | **Characteristics** | | **Reference** |
| --- | --- | --- | --- |
| pET28a+ | Vector used for *mcrB* deletion, Kan^r^ (100 µg/ml) | | Novagen |
| pFLOR06 | *mcrB* deletion plasmid for homologous recombination in CAMSA2038  pET28a+::*OH1+cat+OH2*, Cam^R^ (50 µg/ml), Kan^R^ (100 µg/ml) | | This study |
| **Primer name** | **Sequence** | **Description of amplified region** | **Amplicon size (bp)** |
| cj1421F | TTAATTTAAATCCACCTCCTTTATACC | PolyG tract region in *cj1421* homologue | 600 bp (polyG tract of 9 G’s) |
| cj1421R | AAAATAAAGAAGTGAAAGTAGACGATG |  |  |
| cj1422F | ATCTTCCAAGCAAGTTCAGCA | polyG tract region in *cj1422* homologue | 800 bp (polyG tract of 9 G’s) |
| cj1422R | AGATATTGGTGTGCCTGAGGA |  |  |
| cj1426F | CTACAGGTTGGAAACCAGATA | polyG tract region in *cj1426* homologue | 692 bp (polyG tract of 10 G’s) |
| cj1426R | CGTCTGACTGTCTTGTACAC |  |  |
| FLOR06_OH1_F4 | **CGCGCGGCAGCCAT**AGCCCTATTTTCTTCGCAACTT | Flanking region downstream *mcrB* deletion | 907 bp |
| FLOR06_OH1_R3 | **GTCGCACTGATAAAA**ACTAGATAGAGAAAAAACCATAGGTCA |  |  |
| FLOR06_Cat1_fwd | **TTTTCTCTATCTAGT**TTTTATCAGTGCGACAAACTGG | *cat* amplified for *mcrB* deletion | 753 bp |
| FLOR06_Cat2_rev | **TTTGGGGTGGCTCAG**AGATTTATGATATAGTGGATAGATTTATGATATAATGAGTTA |  |  |
| FLOR06_OH2_F2 | **CTATATCATAAATCT**CTGAGCCACCCCAAATACT | Flanking region upstream *mcrB* deletion | 808 bp |
| FLOR06_OH2_R2 | **GGTGGTGGTGCTCGA**TGAGCAGGGCTGGTATAGG |  |  |
| FLOR06_UP1_fwd | TTGTGTATCAAATTTTGCTTG | Control primers for CAMSA2038*ΔmcrB* | 3596 bp |
| FLOR06_DOWN1_rev | TTTAATGTTCATGAGCAAG |  |  |
| FLOR06_UP2_fwd | TCTTCTAAATTTTCAACACTTAC | Sequencing of CAMSA2038*ΔmcrB* region | - |

**Supplementary Table 4: Detailed *in silico* analysis of DUF262 domain-containing proteins found in CAMSA2002 and CAMSA2038.**

| ***C. jejuni strain*** | **Gene** | **Size (bp)** | **Protein size (aa)** | **Position in genome** | **Putative function** | **Interpro** | **HHpred** |
| --- | --- | --- | --- | --- | --- | --- | --- |
| CAMSA2038 | *DDV75_00040* | 1692 | 563 | 12.653-14.344 | DUF262 domain-containing protein | DUF262 domain in N-terminal, DUF1524 in C-terminal | SspE protein, DNase, 1-561 aa, probability: 100%, E-value 1.3e-50 |
| CAMSA2002 | *DDR89_00040* | 1752 | 583 | 12.642-14.393 | DUF262 domain-containing protein | DUF262 domain in N-terminal | SspE protein, DNase, 1-119 aa, probability: 100%, E-value 2.5e-39 |

**References:**

Fouts, D.E., Mongodin, E.F., Mandrell, R.E., Miller, W.G., Rasko, D.A., Ravel, J., et al. (2005). Major structural differences and novel potential virulence mechanisms from the genomes of multiple *Campylobacter* species. PLoS Biol. *3*, e15. doi: 10.1371/journal.pbio.0030015.

Hansen, V.M., Rosenquist, H., Baggesen, D.L., Brown, S., and Christensen, B.B. (2007). Characterization of *Campylobacter* phages including analysis of host range by selected *Campylobacter* Penner serotypes. BMC Microbiol. *7*, 90. doi: 10.1186/1471-2180-7-90.

Sullivan, M.J., Petty, N.K., and Beatson, S.A. (2011). Easyfig: a genome comparison visualizer. Bioinformatics *27*, 1009-10. doi: 10.1093/bioinformatics/btr039.

Sørensen, M.C.H., Gencay, Y.E., Birk, T., Baldvinsson, S.B., Jäckel, C., Hammerl, J.A., et al. (2015). Primary isolation strain determines both phage type and receptors recognised by *Campylobacter jejuni* bacteriophages. PLoS One *10***,** e0116287. doi: 10.1371/journal.pone.0116287.

Sørensen, M.C.H., Vitt, A., Neve, H., Soverini, M., Ahern, S.J., Klumpp, J., and Brøndsted, L. (2021). *Campylobacter* phages use hypermutable polyG tracts to create phenotypic diversity and evade bacterial resistance. Cell Reports *35*, 109214. doi: 10.1016/j.celrep.2021.109214.
